# Urolithin A Protects Ovarian Reserve Via Inhibiting PI3K/Akt Signaling and Preventing Chemotherapy-Induced Follicle Apoptosis

**DOI:** 10.3390/biology14070829

**Published:** 2025-07-08

**Authors:** Weiyong Wang, Ren Zhou, Yong Ruan, Shuhao Fan

**Affiliations:** 1 Key Laboratory of Animal Genetics, Breeding and Reproduction in the Plateau Mountainous Region, Ministry of Education, Guizhou University, Guiyang 550025, China; wangwy08@outlook.com; 2 College of Animal Science and Technology, Anhui Agricultural University, Hefei 230036, China; zr511823@126.com

**Keywords:** Urolithin A, primordial follicle activation, PI3K/Akt, chemotherapy

## Abstract

Urolithin A, which is a natural gut microbial metabolite, exerts multiple beneficial effects upon supplementation, including prolonging lifespan, mitigating diseases, restoring the quality of aged oocytes and alleviating drug toxicity. The study aims to investigate the ovarian protective role of Urolithin A using a neonatal mouse ovarian in vitro culture and chemotherapy model, with a particular focus on its mechanisms for inhibiting primordial follicle activation and mitigating cyclophosphamide (CY) or 4-hydroperoxy (4-HC)-induced follicle apoptosis. The results showed that Urolithin A significantly decreased the number of growing follicles and downregulated the expression of oocyte growth-related genes (*Gdf9* and *Zp3*) and protein (DDX4), as well as Ki-67 and BrdU-positive signals. Further studies revealed that Urolithin A significantly downregulated the levels of phosphorylated Akt and FOXO3a and decreased the percentage of oocytes with FOXO3a nuclear export. Molecular docking showed a strong binding ability between Urolithin A and its downregulated gene *Pik3cg*. Moreover, Urolithin A significantly decreased CY- and 4-HC-induced increases in cleaved Caspase-3- and PARP1-positive signals. Meanwhile, RNA-seq analysis indicated that Urolithin A significantly downregulated CY-induced expression of DNA damage-related genes (*Trp73* and *Trim29*). In short, Urolithin A inhibits primordial follicle activation by reducing PI3K/Akt signaling reactivity. Furthermore, Urolithin A prevents CY-induced follicle apoptosis. The study provides valuable insights into Urolithin A treatment for chemotherapy-induced infertility.

## Simple Summary

Urolithin A, which is a gut-derived metabolite, demonstrates broad therapeutic potential through lifespan extension, disease mitigation, oocyte quality improvement, and detoxification effects. In the study, Urolithin A inhibited granulosa cell proliferation, downregulated key oocyte growth signals (*Gdf9*, *Zp3*, and DDX4), and blocked PI3K/Akt signaling reactivity, which helps maintain primordial follicle dormancy. It also reduced cyclophosphamide (CY) and 4-hydroperoxy (4-HC)-induced follicle loss by decreasing DNA damage markers (*Trp73* and *Trim29*) and apoptosis signals (Caspase-3 and PARP1). These findings suggest that Urolithin A could help preserve fertility in women undergoing chemotherapy.

## 1. Introduction

Female mammals establish a finite and non-renewable primordial follicle pool around the time of birth, which supplies mature oocytes that are capable of fertilization throughout their reproductive lifespan [1]. In vivo, each primordial follicle has three potential developmental fates—(i) maintaining in a dormant state to preserve the duration of female reproductive lifespan; (ii) undergoing direct attrition from the dormant state, resulting in premature ovarian failure (POF); or (iii) being recruited into the growing follicle pool for subsequent folliculogenesis [2,3]. Under physiological conditions, a small proportion of primordial follicles are recruited into the growing follicle pool during each reproductive cycle, undergoing sequential development and maturation [4]. The molecular regulatory mechanism has been demonstrated to be directly involved in the activation of PI3K/Akt signaling in oocytes and mTOR in granulosa cells [5]. Specifically, activation of mTOR signaling in pre-granulosa cells promotes the expression of the proto-oncogenic receptor tyrosine kinase ligand (KITL), which is upregulated. Secreted KITL binds to the KIT receptors specifically localized on the oocyte membrane, triggering the phosphorylation of tyrosine residue 719 (Y719) on KIT [6]. The phosphorylation event initiates PI3K signaling activation within the oocyte. Phosphatidylinositol 4,5-bisphosphate (PIP2) is catalyzed to phosphatidylinositol 3,4,5-trisphosphate (PIP3) by activated PI3K, thereby recruiting and activating Akt [7,8]. Subsequently, Akt phosphorylates the downstream forkhead box O3 transcription factor (FOXO3a), triggering its nuclear-to-cytoplasmic translocation and thereby relieving the suppression of oocyte growth, which activates primordial follicles [8,9]. Dysregulated primordial follicle activation (either insufficient or excessive) may trigger premature ovarian reserve exhaustion and subsequent premature ovarian insufficiency (POI), which is a reproductive dysfunction marked by imbalanced gonadotropin and estrogen levels [6,10,11]. Thus, precisely controlled primordial follicle activation is vital for preserving a woman’s reproductive lifespan.

In recent years, approximately 10–30% of reproductive-aged women worldwide are affected by POI, which is caused by accumulated genetic and chromosomal variations, environmental pollution, psychological stress, and the widespread use of chemotherapeutic agents driven by the increasing incidence of cancer among younger populations [12,13,14]. As a critical anti-cancer treatment, chemotherapy has been shown to significantly improve survival rates in patients with a wide range of malignancies; however, adverse effects, particularly the premature depletion of ovarian reserves, endocrine dysfunction, and premenopausal infertility, are a threat to survivors [15]. Cyclophosphamide, widely used in treating ovarian cancer, breast cancer, hematologic malignancies, and autoimmune diseases, has been shown to cause significant depletion of primordial follicles through mechanisms involving severe DNA damage, oxidative stress, and ferroptosis [16,17,18]. Cyclophosphamide-caused primordial follicle overactivation via PI3K/Akt signaling significantly contributes to ovarian toxicity [19,20,21]. This pathophysiological mechanism is supported by the complete prevention of ovarian reserve loss through the dual inhibition of both the PI3K/Akt and mTOR pathways, which are key regulators of primordial follicle activation [22]. Oocyte cryopreservation, ovarian tissue cryopreservation, and embryo freezing have become well-established fertility preservation methods for women of reproductive age with urgent reproductive needs who are undergoing chemotherapy [23,24]. However, other consequences of ovarian function loss following chemotherapy, including premature menopause and its associated complications, remain unresolved. Furthermore, pharmacological interventions that preserve ovarian follicles, rather than surgical oophorectomy, may be favored by patients who have completed fertility preservation or do not wish to conceive. Thus, there is a pressing demand to develop safe and effective pharmacological agents as well as practical non-surgical approaches that can protect ovarian reserve and maintain post-chemotherapy endocrine function, which is crucial for women’s health.

Urolithin A is a bioactive metabolite generated by the gut microbiota through the biotransformation of dietary polyphenols, specifically ellagitannins (ETs) and ellagic acid (EA), which are predominantly present in pomegranates, berries, raspberries, and other plant-derived fruits [25,26]. It has drawn significant scientific interest owing to its broad pharmacological properties and potential health benefits, such as its longevity extension and intervention against cardiovascular diseases, neurodegenerative disorders, and metabolic dysfunctions [27]. A clinical evaluation of Urolithin A in a Phase I, randomized, double-blind, placebo-controlled study involving older adults (61–85 years old) confirmed its excellent tolerability and safety [28]. No adverse events were observed following either single oral doses up to 2000 mg or 28- and 120-day repeated administration at daily doses of 1000 mg [28,29]. Pharmacokinetic studies in animal models demonstrated that following the oral administration of Urolithin A, peak concentrations were achieved in blood, urine, and brain tissue at approximately 2, 3, and 4 h post dose, respectively. Subsequently, Urolithin A levels gradually declined and returned to baseline, becoming almost undetectable in urine [30,31,32]. Urolithin A plays pivotal roles in multiple biological processes, including mitophagy activation and regeneration, antioxidant activity, the regulation of cell cycle and apoptosis, metabolic modulation, and anti-inflammatory responses [33]. The execution of these functions is closely associated with Urolithin A-mediated inhibition on PI3K/Akt and mTOR pathways, as well as its activation on AMPK and PGC-1α signaling [34,35]. Interestingly, some studies have also confirmed that Urolithin A improves oocyte quality and extends reproductive lifespan through antioxidant activity and autophagy activation [36,37]. In summary, all of the above-mentioned evidence suggests the possible effects of Urolithin A in the maintenance of primordial follicle dormancy and the prevention of chemotherapy-induced follicle depletion. Therefore, the study explored the potential role of Urolithin A in follicular development using an in vitro ovary culture system coupled with an intraperitoneal administration mouse model, aiming to advance clinical treatments for POI and mitigate chemotherapy-induced ovarian damage.

## 2. Materials and Methods

### 2.1. Animals

All adult healthy ICR male and female mice (about 30–35 g) were purchased from the Guizhou Medical Laboratory Animal Center and were maintained in an individual ventilated cage (IVC) with a suitable temperature (22 ± 1 °C) and humidity (50–70%). Neonatal mice were generated by pairing two-month-old male and female mice in a 1:1 ratio, with the day of delivery being designated as 0.5 days postpartum (dpp). The 3 dpp female mice (about 3–3.5 g) were used for ovarian culture in vitro, whereas chemotherapy models were developed through intraperitoneal injection in 4 dpp mice. All animal protocols were approved by the Institutional Animal Care and Use Committee of Guizhou University (approval number: EAE-GZU-2024-T157).

### 2.2. Mouse Ovary Culture

Ovaries harvested from 3 dpp mice were presented on a Millipore insert and cultured in DMEM/F12 medium supplemented with penicillin–streptomycin solution, as previously reported [9]. To investigate the effect of Urolithin A (HY-100599, 99.82% purity, MCE, Shanghai, China) on primordial follicle activation, we referred to previous studies, adding Urolithin A at final concentrations of 5 μM, 10 μM, and 20 μM to the medium [33,37,38,39]. We performed an in vitro culture of 3-day-old ovaries for a duration of 4 days, with the medium being replaced every 2 days. The vehicle group was cultured in a medium containing 0.1% Dimethyl sulfoxide (DMSO). Furthermore, 1 μM of 4-hydroperoxycyclophosphamide (4-HC—an active metabolite of CY) (HY-117433, MCE, China) was used to establish a chemotherapy drug-induced ovarian injury model in vitro.

### 2.3. Establishment of In Vivo Chemotherapy Model in Newborn Mice

To establish a chemotherapy-induced ovarian injury model, neonatal mice were utilized at 4 dpp. Initially, all 4 dpp mice were injected intraperitoneally with phosphate-buffered saline (PBS) for therapeutic adaptation. Subsequently, the 48 mice were randomly allocated into four experimental groups (12 mice per group) by an independent investigator using the blind method, as follows: a vehicle group (0.1% DMSO), a Urolithin A group, a cyclophosphamide (CY, HY-17420, 99.82% purity, MCE, China) group, and a Urolithin A + CY combination group. At 5 dpp, mice in the vehicle and CY groups were administered DMSO, while those in the Urolithin A and Urolithin A + CY groups received 50 mg/kg Urolithin A via intraperitoneal injection. At 6 dpp, the vehicle group received DMSO, the Urolithin A group was injected with 50 mg/kg Urolithin A, and both the CY and Urolithin A + CY groups were administered 100 mg/kg CY. Notably, mice in the Urolithin A + CY group were pretreated with 50 mg/kg Urolithin A 2 h before CY administration. For phenotypic analysis, ovarian tissues were collected 48 h post treatment, while ovarian samples were harvested 24 h after treatment for molecular mechanism investigations.

### 2.4. Histological and Morphological Analysis

The collected mouse ovaries first underwent fixation in 4% paraformaldehyde (PFA, P1110, Solarbio, Beijing, China) and embedding in paraffin, respectively; then these samples were sectioned into serial slices with a thickness of 5 µm. To perform histological and morphological analyses, these sections were orderly placed in xylene and graded alcohol for deparaffinization and hydration. Finally, they were stained using hematoxylin and were installed in neutral resin for image acquisition. The counting of the number of primordial and growing follicles was conducted according to a previously described method [9]. Only follicles exhibiting discernible nuclei were counted to ensure accuracy and to prevent duplication in counting. Each section was meticulously assessed by two independent evaluators for corroborative purposes.

### 2.5. RNA Sequencing

Total RNA was extracted from the ovaries of 6 dpp mice (3 ovaries per sample, three biological replicates per treatment group), and then mRNA libraries were sequenced using the BGISEQ platform. The raw data were filtered using SOAPnuke (v1.5.2) to obtain clean reads, before being aligned with the reference genes GRCm39 using Bowtie2 (v2.5.4). Finally, the FPKM (fragments per kilobase of exon model per million mapped fragments) was calculated and standardized using RSEM (v1.2.8). Gene expression differences were evaluated using the DESeq2 package in R (v3.21), with candidate genes being defined as those exhibiting a q-value ≤ 0.05 and a log2 fold change ≥ 1.

### 2.6. RNA Isolation and Quantitative Real-Time PCR (qRT-PCR)

Total RNA was extracted from ovaries, and the concentration and purity were detected. Subsequently, 2 μg of RNA was used to generate cDNA using a Reverse Transcription Kit (205311, Qiagen, Germany), and qRT-PCR was performed to quantify mRNA levels. *Actb* was selected as an internal reference to normalize the RT-qPCR data, and the 2^−∆∆CT^ method was used to calculate relative mRNA levels. All the primer sequences for RT-qPCR are listed in Table S1.

### 2.7. Immunofluorescent Staining

Ovarian sections underwent dewaxing and rehydration in xylene and graded alcohols, respectively. Next, these sections were immersed in sodium citrate buffer (pH = 6.0) for antigen retrieval at high temperatures. To block non-specific binding, the sections were exposed to 5% bovine serum albumin (BSA, SW3015, Solarbio, China) for 1 h, before being incubated with primary antibodies at 4 °C overnight. After three washes with PBS, these sections were incubated with Alexa Fluor 488- or 594-conjugated secondary antibodies at 37 °C for 1 h. Finally, nuclear counterstaining was performed using 4,6-diamidino-2-phenylindole (DAPII, C0060, Solarbio, China), followed by image acquisition using a confocal microscope (LSM800, Zeiss, Jena, Germany).

### 2.8. Bromodeoxyuridine (BrdU) Incorporation Assay

The bromodeoxyuridine (BrdU, 550891, Beyotime, China) incorporation assay was used to assess the effect of Urolithin A on ovarian cell proliferation. Briefly, 3-day-old ovaries were cultured for two days, and 10 µM of BrdU was added to the medium 2 h before sample collection, followed by incubation at 37 °C. Finally, the ovaries were harvested and subjected to dehydration, embedding, sectioning, and immunofluorescence staining.

### 2.9. Western Blot Analysis

Protein was isolated from the ovaries using RIPA (P0013B, Beyotime, China) with PMSF (ST506, Beyotime, China) and a protease inhibitor cocktail. In total, 20 µg of denatured protein samples were separated by the 10% SurePAGE^TM^ (M00665, GenScript, Piscataway, NJ, USA) and were then transferred to PVDF membranes (IPVH00010, Millipore, Germany). The membranes were blocked in TBST with 5% BSA for 1 h before being incubated with primary antibodies overnight at 4 °C. After three washes with PBST, the membranes were treated with secondary antibodies for 1 h. Finally, the bands were visualized in the Tanon 5200 imaging system with ECL chromogenic solutions (ESL003, Biolight Biotech, Guangzhou, China). The grayscale values of these bands were quantified using ImageJ software (v1.8.0.112) and were then normalized by β-actin. All antibody information is available in Table S2.

### 2.10. Statistical Analysis

Each experiment was conducted with at least three independent biological replicates, and data are displayed as means ± standard deviations (SDs). Significance was performed using a Student’s *t*-test after assessment of a normal distribution, and the data were visualized with GraphPad Prism 9. Differences were defined as *p* < 0.05 (* *p* < 0.05, ** *p* < 0.01, and *** *p* < 0.001).

## 3. Results

### 3.1. Urolithin A Maintains Primordial Follicle Dormancy In Vitro

**Figure 1:**
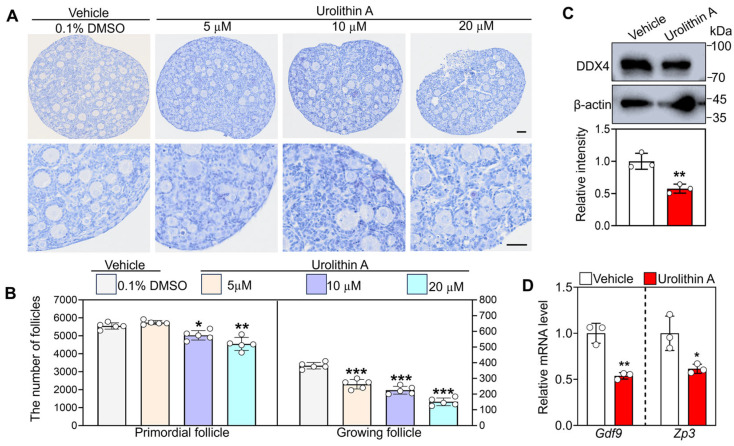
Urolithin A promotes mouse primordial follicle activation. (**A**) Ovarian morphological examination by hematoxylin staining. (**B**) The number of primordial and growing follicles. (**C**) Western blot analysis of DDX4 protein levels. (**D**) RT-qPCR analysis of *Gdf9* and *Zp3* mRNA levels. Both the vehicle- and Urolithin A-treated groups contained 0.1% DMSO. In each experiment, *n* ≥ 3 biological replicates. Bars indicate the mean ±SD. A two-sided Student’s *t*-test was used to determine *p*-values (* *p* < 0.05, ** *p* < 0.01, and *** *p* < 0.001). Scale bar = 50 µm.

To evaluate the effects of Urolithin A on primordial follicle activation, the 3 dpp mouse ovaries were cultured in vitro for 4 days, either in vehicle or Urolithin A. Hematoxylin staining and follicle counting showed that 5 µM Urolithin A caused a significant decrease in the number of growing follicles relative to the vehicle (381 ± 18.28 vs. 263 ± 26.94), but exerted no significant effect on the primordial follicle number (Figure 1A,B). However, treatment of Urolithin A (10 and 20 µM) exacerbated follicle atresia in a concentration-dependent manner, accompanied by a marked decrease in the primordial and growing follicle number (Figure 1A,B). Based on these findings, 5 µM was determined to be the optimal concentration for further experiments. As markers of oocyte growth, the mRNA levels of growth differentiation factor 9 (*Gdf9*) and zona pellucida 3 (*Zp3*), along with the protein levels of DEAD-box helicase 4 (DDX4), were significantly reduced in ovaries following Urolithin A treatment compared to vehicle groups (Figure 1C,D). These results indicate that Urolithin A treatment inhibits oocyte growth and the activation of primordial follicles.

### 3.2. Urolithin A Treatment Inhibits Granulosa Cell Proliferation

**Figure 2:**
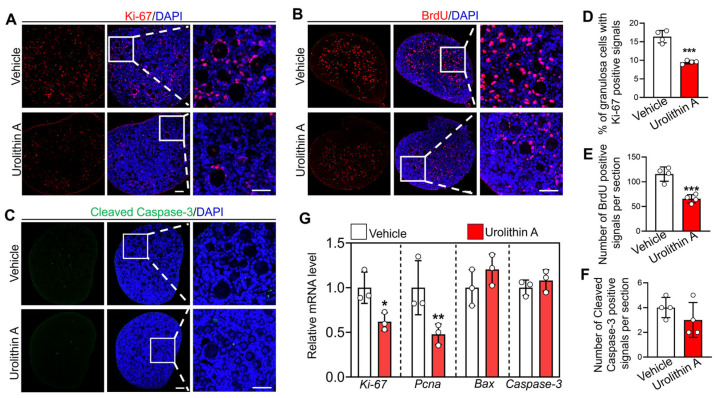
Urolithin A inhibits the proliferation of granulosa cells. (**A**–**C**) The immunofluorescence staining of Ki-67, BrdU, and cleaved Caspase-3. (**D**–**F**) The quantitative analysis of Ki-67-, BrdU-, and cleaved Caspase-3-positive signals. (**G**) RT-qPCR analysis of *Ki-67*, *Pcna*, *Bax,* and *Caspase-3* mRNA levels. Both the vehicle- and Urolithin A-treated groups contained 0.1% DMSO. In each experiment, *n* ≥ 3 biological replicates. Bars indicate the mean ± SD. A two-sided Student’s *t*-test was used to determine *p*-values (* *p* < 0.05, ** *p* < 0.01, and *** *p* < 0.001). Scale bar = 50 µm.

Since granulosa cell proliferation is an indispensable event in primordial follicle activation, the effects of Urolithin A on granulosa cell proliferation were further investigated. BrdU incorporation, immunofluorescence staining, and statistical analysis demonstrated that Urolithin A treatment markedly reduced the percentages of granulosa cells with Ki-67 positive staining (16.37% ± 1.44 vs. 9.52% ± 0.41), as well as the number of BrdU-labeled somatic cells (115.5 ± 12.46 vs. 75.6 ± 7.23), relative to the vehicle groups (Figure 2A,B,D,E). Consistently, Urolithin A notably decreased the mRNA levels of proliferating cell nuclear antigen (*Pcna*) and *Ki-67* compared to the vehicle groups (Figure 2G). Importantly, Urolithin A treatment showed no significant effects on either the mRNA levels of Bax and Caspase-3 or the positive staining of cleaved Caspase-3 protein (Figure 2C,F,G). In summary, these findings reveal that Urolithin A maintains mouse primordial follicle dormancy in vitro by inhibiting granulosa cell proliferation without inducing apoptosis.

### 3.3. Urolithin A Maintains Primordial Follicle Dormancy Via Inhibiting PI3K/Akt Signaling

**Figure 3:**
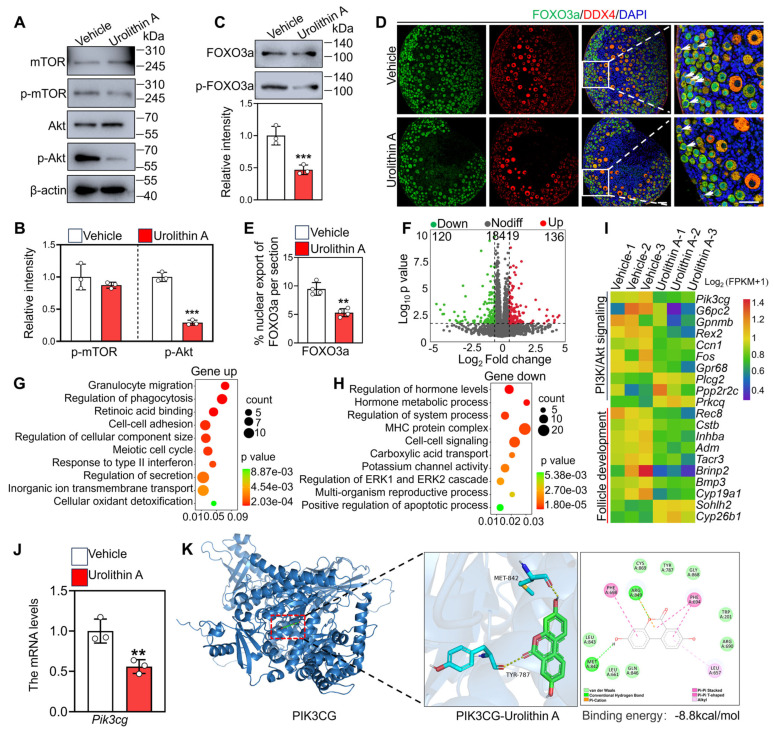
Urolithin A inhibits the PI3K/Akt pathway in mouse ovaries. (**A**–**C**) Western blot analysis of p-mTOR, p-Akt, and p-FOXO3a protein levels. (**D**) Immunofluorescence co-staining of FOXO3a (green) and DDX4 (red). The arrow points to oocytes with FOXO3a nuclear export. (**E**) The percentage of oocytes with FOXO3a nuclear export. (**F**) Volcano diagram illustrating the transcripts with differential expression between vehicle- and Urolithin A-treated ovaries. (**G**,**H**) Bubble chart illustrating the GO terms enriched by downregulated and upregulated transcripts in Urolithin A-treated ovaries. (**I**) Heatmaps illustrating a group of differential transcripts involved in the indicated biological processes in vehicle- and Urolithin A-treated ovaries. (**J**) qRT-PCR validation of *Pik3cg* mRNA levels. (**K**) Molecular docking reveals the binding capability between Urolithin A and PI3KCG. Both the vehicle- and Urolithin A-treated groups contained 0.1% DMSO. Bars indicate the mean ±SD. A two-sided Student’s *t*-test was used to determine *p*-values (** *p* < 0.01 and *** *p* < 0.001). Scale bar = 50 µm.

PI3K/Akt in oocytes and mTOR in pre-granulosa cells are crucial pathways for primordial follicle activation. Western blot analysis showed that Urolithin A treatment significantly downregulated the protein levels of phosphorylated Akt (p-Akt) in cultured neonatal mouse ovaries relative to the vehicle group, while exerting no significant effect on phosphorylated mTOR (p-mTOR) levels (Figure 3A,B), suggesting that Urolithin A preferentially targets the PI3K/Akt signaling pathway rather than mTOR in the ovaries. Furthermore, compared with the vehicle group, Urolithin A treatment downregulated the phosphorylated FOXO3a (marker of primordial follicle activation) protein levels and significantly decreased the proportion of oocytes exhibiting FOXO3a nuclear export (9.48% ± 0.96 vs. 5.30% ± 0.61) (Figure 3C–E).

Comprehensive transcriptome profiling via RNA sequencing was performed to indicate the molecular mechanisms underlying Urolithin A-mediated primordial follicle dormancy. The results showed that 256 transcripts (including 136 upregulated and 120 downregulated) exhibited differential expression between the Urolithin A treatment group and the vehicle group (Figure 3F). GO analysis showed that the upregulated transcripts were involved in terms such as retinoic acid binding, cell–cell adhesion, and meiotic cell cycle (Figure 3G). In contrast, the downregulated genes mainly participated in PI3K/Akt signaling, follicle development, the regulation of hormone levels, and cell–cell signaling (Figure 3H,I). Notably, the downregulation of *Pik3cg*, *Ccn1*, *Gpr68*, *Prkcq,* and *Plcg2* could directly inhibit the reactivity of PI3K/Akt signaling (Figure 3I,J). Ulteriorly, molecular docking results indicated that Urolithin A exhibited strong binding affinity to PIK3CG (binding energy = −8.8 kcal/mol) (Figure 3K). Altogether, these results indicated that Urolithin A inhibits primordial follicle activation via PI3K/Akt signaling.

### 3.4. Urolithin A Reverses Cyclophosphamide-Induced Loss of Primordial Follicles

**Figure 4:**
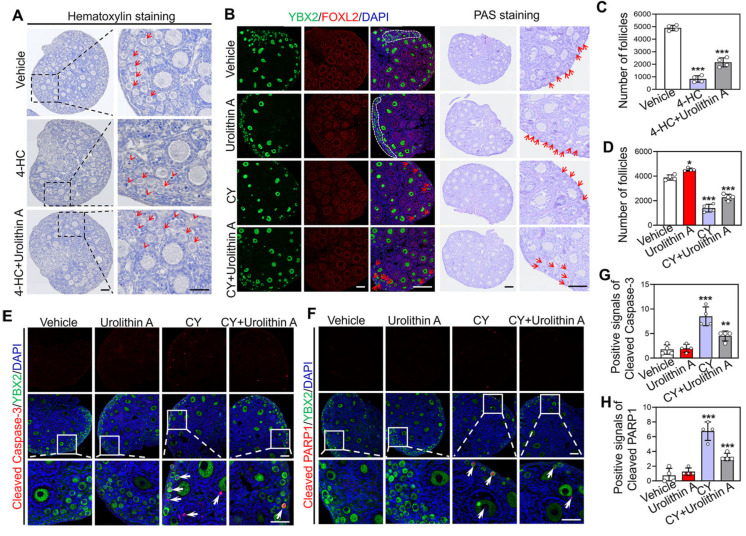
Urolithin A protects 4-HC and CY-induced follicle apoptosis. (**A**) Morphological examination of ovaries treated with the vehicle, 4-HC, and 4-HC+ Urolithin A in vitro. The arrowhead points to apoptotic follicles, while the arrow points to primordial follicles. (**B**) Morphological examination of ovaries treated with the vehicle, Urolithin A, CY, and CY + Urolithin A in vivo. The arrowhead and irregular box point to primordial follicles. (**C**,**D**) The counting of several primordial follicles. (**E**,**F**) Immunofluorescence staining of cleaved Caspase-3 and cleaved PARP1. The arrowhead points to apoptotic signals. (**G**,**H**) The counting of cleaved Caspase-3- and cleaved PARP1-positive signals. Both the vehicle-, Urolithin A-, CY-, and CY + Urolithin A-treated groups contained 0.1% DMSO. The red arrowhead indicates an atretic follicle, while the red arrow indicates primordial follicle. The white arrow indicates the apoptosis signal. Bars indicate the mean ±SD. A two-sided Student’s *t*-test was used to determine *p*-values (* *p* < 0.05, ** *p* < 0.01 and *** *p* < 0.001). Scale bar = 50 µm.

Given the roles of Urolithin A in maintaining primordial follicle dormancy, inhibiting PI3K/Akt activity, and exerting antioxidant and anti-inflammatory effects, we are considering its potential as a protective agent for the survival of primordial follicles during chemotherapy. Hematoxylin staining and follicle counting showed that 4-HC significantly caused primordial follicle loss in cultured neonatal mouse ovaries relative to the vehicle group (4897.5 ± 186.23 vs. 826.25 ± 224.68) (Figure 4A,C). However, the combination therapy of 4-HC and Urolithin A significantly increased the number of primordial follicles relative to the 4-HC treatment group (826.25 ± 224.68 vs. 2153.75 ± 313.20) (Figure 4A,C). Consistent with these in vitro findings, intraperitoneal injection of Urolithin A significantly increased the loss in the number of primordial follicles induced by CY in vivo (2275 ± 216.71 vs. 1392.5 ± 273.89) (Figure 4B,D). Furthermore, Urolithin A markedly reduced the positive signals of cleaved Caspase-3 (8.5 ± 1.66 vs. 4.5 ± 0.87) and PARP1 (6.75 ± 1.01 vs. 3.25 ± 0.43), which were caused by CY injection (Figure 4E–H). These results indicate that Urolithin A can reverse CY-induced primordial follicle apoptosis.

### 3.5. Transcriptome Reveals the Protective Effect of Urolithin A on Cyclophosphamide Induced Ovarian Damage

**Figure 5:**
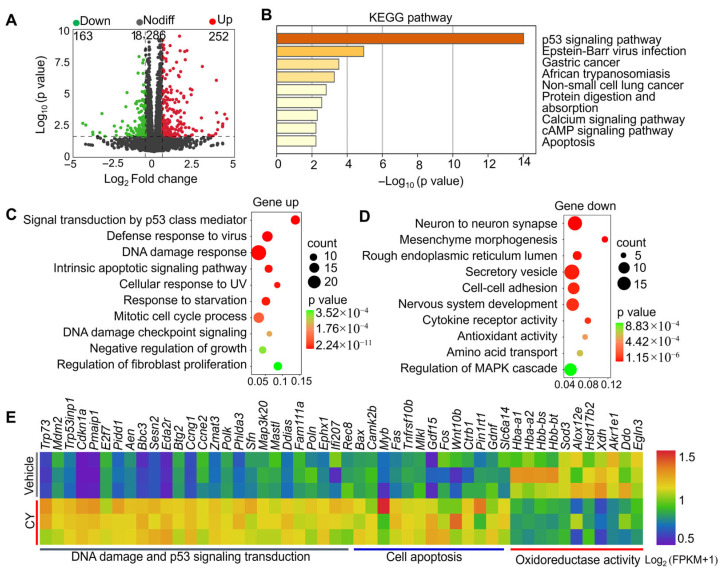
Transcriptomic analysis of cyclophosphamide-induced follicle apoptosis. (**A**) Volcano diagram illustrating the transcripts with differential expression between vehicle- and CY-treated ovaries. (**B**) Bar charts illustrating the KEGG terms enriched by differential transcripts in CY-treated ovaries. (**C**,**D**) Bubble chart illustrating the GO terms enriched by downregulated and upregulated transcripts in CY-treated ovaries. (**E**) Heatmaps illustrating a group of upregulated and downregulated transcripts involved in the indicated biological processes in CY-treated ovaries. Both the vehicle- and CY-treated groups contained 0.1% DMSO.

To further elucidate the potential mechanism by which Urolithin A reverses CY-induced follicle apoptosis, the ovaries from mice injected with vehicle, CY, and CY + Urolithin A were subjected to RNA-seq analysis. Compared with vehicle groups, 415 transcripts (including 252 upregulated and 163 downregulated) exhibited differential expression in CY-treated ovaries (Figure 5A). KEGG enrichment analysis showed that these differentially expressed genes were involved in biological processes such as the p53 signaling pathway, the calcium and cAMP signaling pathway, and apoptosis (Figure 5B). Consistently, GO enrichment showed that the upregulated transcripts participate in processes such as DNA damage and p53 signaling transduction, cell apoptosis, and the negative regulation of growth (Figure 5C,E). Particularly, the upregulation of *Trp73*, *Mdm2*, *Cdkn1a*, *Bax,* and *Fas* could directly result in the apoptosis/death of the follicle (Figure 5E). In contrast, the downregulated genes mainly participated in secretory vesicle, cell–cell adhesion, and oxidoreductase activity (Figure 5D,E). These results suggest that increased DNA damage and reduced antioxidant capacity may be the principal reasons for the cyclophosphamide-induced apoptosis of the follicle.

**Figure 6:**
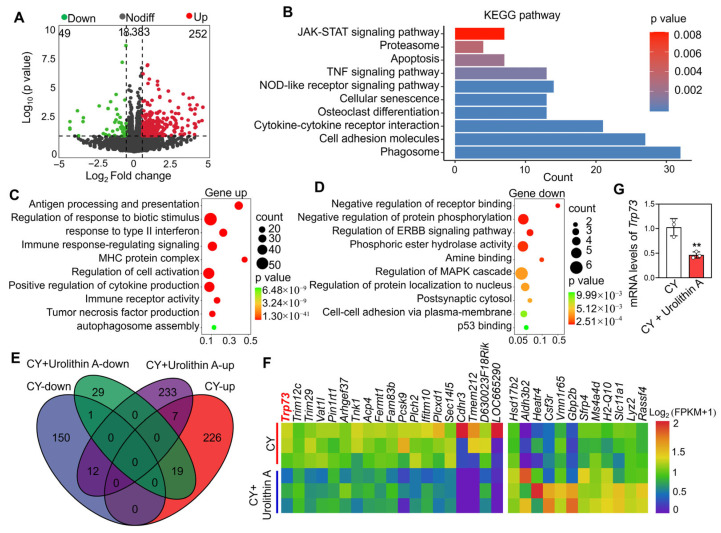
Transcriptomic analysis of the effect of Urolithin A on cyclophosphamide-induced follicle apoptosis. (**A**) Volcano diagram illustrating the transcripts with differential expression between CY and CY + Urolithin A-treated ovaries. (**B**) Bar charts illustrating the KEGG terms enriched by differential transcripts in CY + Urolithin A-treated ovaries. (**C**,**D**) Bubble chart illustrating the GO terms enriched by downregulated and upregulated transcripts in CY + Urolithin A-treated ovaries. (**E**) Venn diagram showing the relationship of differential transcripts in CY- and CY + Urolithin A-treated ovaries. (**F**) Heatmaps illustrating a group of transcripts that were upregulated in CY-induced ovaries but suppressed by CY + Urolithin A co-treatment, alongside another group of transcripts that were downregulated in CY-induced ovaries but upregulated by CY + Urolithin A co-treatment. (**G**) qRT-PCR validation of *Trp73* mRNA levels. Both the CY- and CY + Urolithin A-treated groups contained 0.1% DMSO. Bars indicate the mean ±SD. A two-sided Student’s *t*-test was used to determine *p*-values (** *p* < 0.01).

Subsequently, the transcriptional changes were compared between ovaries treated with CY + Urolithin A and those treated with CY alone. The results showed that 301 transcripts (including 252 upregulated and 49 downregulated) exhibited differential expression in CY + Urolithin A-treated ovaries (Figure 6A). KEGG enrichment revealed that differentially expressed genes participated in biological processes including the phagosome, cell adhesion molecules, and the TNF signaling pathway, as well as apoptosis (Figure 6B). GO enrichment showed that upregulated genes mainly involved in terms related to inflammation, such as antigen processing and presentation, the regulation of responses to biotic stimuli, and immune receptor activity, while the downregulated transcripts-controlled processes such as p53 binding, the negative regulation of protein phosphorylation, and the regulation of the ERBB signaling pathway (Figure 6C,D). Furthermore, transcriptomic intersection analysis revealed that 12 transcripts downregulated in CY-treated ovaries were upregulated after Urolithin A treatment, including the pro-folliculogenic factors *Hsd17b2* and *Aldh3b2* (Figure 6E,F). Importantly, Urolithin A downregulated 19 transcripts in ovaries that had been upregulated by CY treatment, including *Trp73*, which is a key target of chemotherapy-induced primordial follicle apoptosis (Figure 6E,F). Ulteriorly, qRT-PCR analysis also showed that Urolithin A treatment decreased the *Trp73* mRNA levels induced by CY (Figure 6G). These findings suggest that Urolithin A could protect follicles from CY toxicity by reducing DNA damage.

## 4. Discussion

Urolithin A is a secondary metabolite with significant clinical potential, demonstrating beneficial effects in disease treatment and lifespan extension. This study demonstrates that Urolithin A inhibits primordial follicle activation through PI3K/Akt signaling. Furthermore, Urolithin A prevents CY-induced primordial follicle loss in mice.

In our study, Urolithin A, as a secondary metabolite derived from fruits and vegetables, has been demonstrated to inhibit primordial follicle activation in mice at appropriate concentrations. The underlying mechanism involves the reduced activity of PI3K/Akt signaling, which is a classical signaling route that participates in primordial follicle activation. Extensive research has demonstrated that Urolithin A can inhibit signaling pathways including mTOR, PI3K/Akt, and ERK in T cells, nerve cells, and tumor cells [40,41,42], highlighting its therapeutic potential in treating tumors and neurological disorders. These findings further corroborate our conclusion that Urolithin A suppresses PI3K/Akt activity in mouse ovaries. Moreover, Urolithin A treatment induces cell cycle arrest of colorectal cancer cells, as well as inhibiting cell proliferation, migration, and invasion in cardiac fibroblasts and primary multiple myeloma cells [43,44,45]. These findings align with our demonstration of Urolithin A-mediated proliferation inhibition in ovarian granulosa cells. Therefore, Urolithin A treatment may directly inhibit pre-granulosa cell proliferation in primordial follicles, thereby suppressing primordial follicle activation. Future studies should explore combination therapies of Urolithin A with PI3K/Akt or KIT inhibitors to fully elucidate the mechanisms underlying the Urolithin A-mediated inhibition of primordial follicle activation. Interestingly, transcriptomic profiling revealed the significant downregulation of *Pik3cg* (PI3Kγ/p110γ) in Urolithin A-treated ovaries. Complementary molecular docking studies demonstrated strong binding interactions between Urolithin A and PIK3CG, potentially explaining the observed attenuation of PI3K/Akt signaling. Consistent with this, prior research has established that the pharmacological inhibition of PI3Kγ/p110γ with small-molecule agents potently attenuates PI3K/Akt signaling in human and murine leukemia cells, macrophages, and T cells [46,47,48]. Taken together, our study reveals that Urolithin A-mediated primordial follicle dormancy via PI3K/Akt pathway inhibition likely involves *Pik3cg* downregulation. Further biophysical validation using surface plasmon resonance and isothermal titration calorimetry will be crucial to confirm these interactions. In short, this evidence suggests that the appropriate supplementation of Urolithin A in females may help protect ovarian reserves.

The fertility of childbearing-age women with cancer is threatened by chemotherapy, which induces excessive primordial follicle activation and DNA damage-triggered apoptosis [49,50]. Recent studies have shown that targeted interventions, including genetic knockout, the pharmacological inhibition of the PI3K/Akt and mTOR signaling pathways, and antioxidant treatment, can partially preserve ovarian reserves in mice undergoing chemotherapy [22,51,52]. The present study demonstrates that Urolithin A treatment effectively reversed the cyclophosphamide-caused depletion of primordial follicles, both in vivo and in vitro (using 4-hydroperoxycyclophosphamide—4-HC), while concurrently suppressing apoptotic signaling. As a known inhibitor of the PI3K/Akt and mTOR pathways, as well as a well-established mitophagy activator [27,53,54], Urolithin A may reduce chemotherapy-caused follicle apoptosis/death through multiple mechanisms, including the inhibition of primordial follicle activation, the enhancement of autophagy, and improving antioxidant capacity. These proposed mechanisms were supported at the transcriptional level by the ability of Urolithin A to reverse the cyclophosphamide-induced downregulation of antioxidant factors in ovaries, such as *Aldh3b2* and *Slc11a1*. The present study also supports the previous standpoint that blocking the key signaling pathways involved in primordial follicle activation during chemotherapy can effectively maintain ovarian reserve function. Furthermore, the p53 family members (p53, p63, and p73) act as central mediators of chemotherapy-induced DNA damage and apoptosis in primordial follicles [55,56]. The genetic ablation or pharmacological suppression of these genes, or their upstream regulator *Check1/2*, can effectively protect primordial follicles from chemotherapy-induced damage [51,57,58,59]. In the present study, Urolithin A treatment significantly attenuated cyclophosphamide-induced *Trp73* upregulation, suggesting that its protective effects against follicle depletion are mediated through DNA damage reduction. Similarly to Urolithin A, quercetin can also alleviate cyclophosphamide-induced primordial follicle depletion in mice by inhibiting the PI3K/Akt/Foxo3a pathway, but it has low bioavailability and may induce DNA damage [60,61,62]. These findings indicate that Urolithin A could serve as a potential therapeutic drug for protecting the ovarian reserves of female cancer patients undergoing chemotherapy.

However, it should be noted that while the study demonstrates that Urolithin A protects primordial follicles in mice, its efficacy in preserving human ovarian reserve has not been confirmed due to the absence of clinical trials. Further in vitro culture of human ovarian fragments is needed to determine the role of Urolithin A in ovarian reserve preservation. Urolithin A was confirmed to inhibit PI3K/Akt activity in this study, but its additional molecular mechanisms, optimal dosing, and long-term safety (particularly regarding normal follicle development) need further study. Furthermore, our study focused solely on cyclophosphamide-induced ovarian injury. Since different chemotherapeutic agents (e.g., cisplatin and doxorubicin) may damage follicles through distinct mechanisms, the efficacy of Urolithin A as a broad-spectrum ovarian protectant requires further validation.

## 5. Conclusions

In summary, in vitro, in vivo, and transcriptomic analyses collectively demonstrate that Urolithin A maintains primordial follicle dormancy via suppressing PI3K/Akt signaling and alleviating CY-induced follicle apoptosis. As a well-characterized secondary metabolite with established safety in humans, Urolithin A may serve as a promising therapeutic candidate for addressing infertility in women with POI, advanced maternal age, or chemotherapy-induced ovarian damage.

## Data Availability

The RNA-seq raw data have been stored in the GEO database with approval number GSE299728.

## Supplementary Materials

The following supporting information can be downloaded at: https://www.mdpi.com/article/10.3390/biology14070829/s1, Figure S1: The original Western Blots; Table S1: Primers for qRT-PCR used in this study; Table S2: List of primary antibodies used in immune detection in this study.

## Abbreviations

Akt	Protein kinase B
Aldh3b2	Aldehyde dehydrogenase 3 family, member B2
AMPK	AMP-activated protein kinase
Bax	BCL-2-associated X protein
BrdU	Bromodeoxyuridine
BSA	Bovine serum albumin
Caspase-3	Cysteinyl aspartate specific proteinase
Ccn1	Cellular communication network factor 1
Cdkn1a	Cyclin-dependent kinase inhibitor 1A
CY	cyclophosphamide
DAPI	4,6-Diamidino-2-phenylindole
DDX4	DEAD-box helicase 4
DMSO	Dimethyl sulfoxide
dpp	Days postpartum
EA	Ellagic acid
ETs	Ellagitannins
Fas	Factor-related apoptosis
FOXO3a	Forkhead Box O3a
Gdf9	Growth differentiation factor 9
GO	Gene ontology
Grp68	G protein-coupled receptor 68
Hsd17b2	hydroxysteroid 17-beta dehydrogenase 2
KEGG	Kyoto encyclopedia of genes and genomes
KITL	Proto-oncogenic receptor tyrosine kinase ligand
Mdm2	Mouse double minute 2 homolog
mTOR	Mammalian target of rapamycin
PARP1	poly (ADP-ribose) polymerase family, member 1
PCNA	Proliferating cell nuclear antigen
PFA	Paraformaldehyde
PGC-1α	Peroxisome proliferator-activated receptor γ coactivator α
PI3K	Phosphatidylinositol-3-kinase
PIP2	Phosphatidylinositol 4,5-bisphosphate
PIP3	Phosphatidylinositol 3,4,5-trisphosphate
PIK3CG	Phosphatidylinositol-4,5-bisphosphate 3-kinase catalytic subunit γ
Plcg2	Phospholipase C gamma 2
PMSF	Phenylmethanesulfonyl fluoride
POF	Premature ovarian failure
POI	Premature ovarian insufficiency
Prkcq	Protein kinase C theta
PVDF	Polyvinylidene fluoride
RIPA	Radio Immunoprecipitation assay lysis buffer
TBST	Tris buffer saline Tween-20
Trim29	Tripartite motif-containing 29
Trp73	Transformation related-protein 73
Zp3	Zona pellucida 3
4-HC	4-Hydroperoxycyclophosphamide
