# Supplementary material for: Urolithin A Protects Ovarian Reserve Via Inhibiting PI3K/Akt Signaling and Preventing Chemotherapy-Induced Follicle Apoptosis
Source: Biology (Basel). 2025 Jul 8;14(7):829. doi: 10.3390/biology14070829 (PMC12292322; doi:10.3390/biology14070829)
Supplement: Supplementary file 1 [file biology-14-00829-s001.zip › Supplementary information.pdf]

Urolithin A Protects Ovarian Reserve Via Inhibiting PI3K/Akt Signaling and Preventing Chemotherapy-Induced Follicle Apoptosis

Weiyong Wang[1,†], Ren Zhou[2,†], Yong Ruan[1,*], Shuhao Fan[2,*]

[1] Key Laboratory of Animal Genetics, Breeding and Reproduction in the Plateau Mountainous Region, Ministry of Education, Guizhou University, Guiyang 550025, China.

[2] College of Animal Science and Technology, Anhui Agricultural University, Hefei 230036, China.

*Corresponding author: Shuhao Fan, College of Animal Science and Technology, Anhui Agricultural University, Hefei 230036, China. E-mail: shuhaowudi@163.com; Yong Ruan, Key Laboratory of Animal Genetics, Breeding and Reproduction in the Plateau Mountainous Region, Ministry of Education, Guizhou University, Guiyang 550025, China. E-mail: yruan@gzu.edu.cn.

† These authors contributed equally to this work.

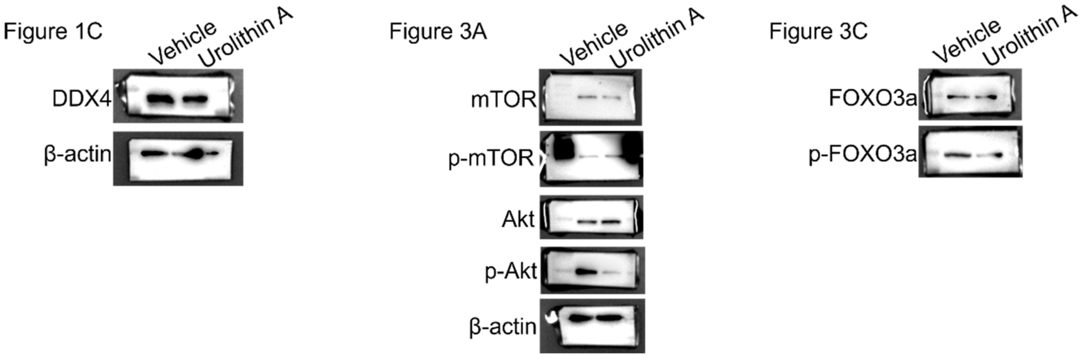

Figure S1. The original Western Blots

Table S1. Primers for qRT–PCR used in this study.

Genes	*Forwards (5′-3′)*	*Backwards (5′-3′)*
Bax	*TTTCATCCAGGATCGAGCAGG*	*GCAAAGTAGAAGAGGGCAACCAC*
Caspase-3	*CCGGTTACTATTCCTGGAGA*	*TAACACGAGTGAGGATGTGC*
Gdf9	*TCTTAGTAGCCTTAGCTCTCAGG*	*TGTCAGTCCCATCTACAGGCA*
Ki-67	*ATCATTGACCGCTCCTTTAGGT*	*GCTCGCCTTGATGGTTCCT*
Pcna	*CGGCGTGAACCTGCAGAGCA*	*GGTTGCGGTCGCAGCGGTAT*
Actb	*GGCTGTATTCCCCTCCATCG*	*CCAGTTGGTAACAATGCCATGT*
Pik3cg	*CGAGAGTGTCGTCACAGTGTC*	*TGTTCGCTTCCACACAAACAG*
Trp73	*GCACCTACTTTGACCTCCCC*	*GCACTGCTGAGCAAATTGAAC*
Zp3	*CCTCAGGACTAACCGTGTGGA*	*CCATCAGGCGAAGAGAGAAAG*

Table S2. List of primary antibodies used in immune detection in this study.

Antibody	*Catalog Code*	*Source*	*Host*	*Dilution*	
				IF	*WB*
Akt	*4691*	*Cell Signaling Technology*	*Rabbit*	*—*	*1:1000*
p-Akt	*4060*	*Cell Signaling Technology*	*Rabbit*	*1:200*	*1:1000*
BrdU	*ab1893*	*Abcam*	*Sheep*	*1:200*	*—*
Cleaved Caspase-3	*9664*	*Cell Signaling Technology*	*Rabbit*	*1:50*	*—*
DDX4	*ab27591*	*Abcam*	*Mouse*	*1:200*	*1:1000*
FOXO3a	*12829*	*Cell Signaling Technology*	*Rabbit*	*1:200*	*1:1000*
p-FOXO3a	*ab26649*	*Abcam*	*Rabbit*	*—*	*1:1000*
Ki-67	*9129s*	*Cell Signaling Technology*	*Rabbit*	*1:300*	*—*
mTOR	*2972*	*Cell Signaling Technology*	*Rabbit*	*—*	*1:1000*
p-mTOR	*2971*	*Cell Signaling Technology*	*Rabbit*	*—*	*1:1000*
β-actin	*4967*	*Cell Signaling Technology*	*Rabbit*	*—*	*1:1000*

IF: Immunofluorescence; WB: Western blotting
